# Influence of Donor-Specific Characteristics on Cytokine Responses in H3N2 Influenza A Virus Infection: New Insights from an Ex Vivo Model

**DOI:** 10.3390/ijms252010941

**Published:** 2024-10-11

**Authors:** Chung-Guei Huang, Ming-Ju Hsieh, Yi-Cheng Wu, Po-Wei Huang, Ya-Jhu Lin, Kuo-Chien Tsao, Shin-Ru Shih, Li-Ang Lee

**Affiliations:** 1 Department of Laboratory Medicine, Chang Gung Memorial Hospital, Linkou Branch, Taoyuan 33305, Taiwan; joyce@cgmh.org.tw (C.-G.H.); mito1005@cgmh.org.tw (P.-W.H.); niny@cgmh.org.tw (Y.-J.L.); kctsao@cgmh.org.tw (K.-C.T.); srshih@mail.cgu.edu.tw (S.-R.S.); 2 Department of Medical Biotechnology and Laboratory Science, Chang Gung University, Taoyuan 33302, Taiwan; 3 Research Center for Emerging Viral Infections, Chang Gung University, Taoyuan 33302, Taiwan; 4 Division of Thoracic and Cardiovascular Surgery, Department of Surgery, Chang Gung Memorial Hospital, Linkou Branch, Taoyuan 33305, Taiwan; hsiehmj2@gmail.com (M.-J.H.); expert96@cgmh.org.tw (Y.-C.W.); 5 School of Medicine, College of Medicine, Chang Gung University, Taoyuan 33302, Taiwan; 6 School of Medicine, College of Life Science and Medicine, National Tsing Hua University, Hsinchu 300044, Taiwan; 7 Department of Otorhinolaryngology, Head and Neck Surgery, Chang Gung Memorial Hospital, Linkou Main Branch, Taoyuan 33305, Taiwan

**Keywords:** inflammasome, cytokine, influenza A virus, ex vivo infection, Toll-like receptor, programmed death 1/programmed death-ligand 1, lung cancer, cigarette smoking, diabetes mellitus

## Abstract

Influenza A virus (IAV) is known for causing seasonal epidemics ranging from flu to more severe outcomes like pneumonia, cytokine storms, and acute respiratory distress syndrome. The innate immune response and inflammasome activation play pivotal roles in sensing, preventing, and clearing the infection, as well as in the potential exacerbation of disease progression. This study examines the complex relationships between donor-specific characteristics and cytokine responses during H3N2 IAV infection using an ex vivo model. At 24 h post infection in 31 human lung explant tissue samples, key cytokines such as interleukin (IL)-6, IL-10, tumor necrosis factor-alpha (TNF-α), and interferon-gamma (IFN-γ) were upregulated. Interestingly, a history of lung cancer did not impact the acute immune response. However, cigarette smoking and programmed death-ligand 1 (PD-L1) expression on macrophages significantly increased IL-2 levels. Conversely, age inversely affected IL-4 levels, and diabetes mellitus negatively influenced IL-6 levels. Additionally, both diabetes mellitus and programmed cell death protein 1 (PD-1) expression on CD3^+^/CD4^+^ T cells negatively impacted TNF-α levels, while body mass index was inversely associated with IFN-γ production. Toll-like receptor 2 (TLR2) expression emerged as crucial in mediating acute innate and adaptive immune responses. These findings highlight the intricate interplay between individual physiological traits and immune responses during influenza infection, underscoring the importance of tailored and personalized approaches in IAV treatment and prevention.

## 1. Introduction

Influenza viruses, particularly influenza A virus (IAV), pose a significant global health threat due to their pandemic potential [1]. Capable of causing severe respiratory illnesses, these viruses can lead to conditions ranging from acute lung injury to acute respiratory distress syndrome (ARDS) and, in severe cases, death [2]. In the United States, it is estimated that 30–40% of hospitalized patients with laboratory-confirmed influenza develop acute pneumonia [3,4]. Annually, influenza—especially the H1N1 and H3N2 IAV strains—causes significant morbidity and mortality [5], highlighting the need for effective disease management and a deeper understanding of its pathophysiology.

At the forefront of the body’s defense against IAV infection are inflammasomes, multi-protein intracellular complexes crucial in the innate immune system. These complexes play a pivotal role by activating inflammatory responses and facilitating the release of pro-inflammatory cytokines such as interleukin (IL)-1β and IL-18 [6,7]. The activation of inflammasomes is triggered by various stimuli including infectious agents, environmental irritants, and cellular stress, leading to pyroptosis—a form of cell death that eliminates pathogen-infested cells and alerts the immune system to potential threats [8,9].

Recent research has broadened our understanding of the role of inflammasomes beyond traditional views. It is now known that they regulate key biological processes such as autophagy, metabolism, and the eicosanoid storm—critical for modulating inflammation and immune responses [10]. Furthermore, the excessive activation of inflammasomes can lead to cytokine storms, a severe immune reaction characterized by an overactive inflammatory response that can damage organs rather than resolve infections [11,12].

During an IAV infection, the activation of inflammasomes in macrophages and dendritic cells can release large amounts of cytokines and chemokines including IL-1β, IL-2, IL-4, IL-6, IL-10, tumor necrosis factor-alpha (TNF-α), and interferon-gamma (IFN-γ) [13,14,15]. These mediators are crucial for coordinating the body’s defense but can also lead to detrimental outcomes if produced excessively [16].

Macrophages, essential components of the innate immune system [17], play a key role in detecting and engulfing the virus. The interaction between viral pathogen-associated molecular patterns (PAMPs) or damage-associated molecular patterns (DAMPs) and inflammasomes in these cells is crucial, especially in the lungs, where an excessive inflammatory response can result in ARDS [18,19,20].

T cells play a pivotal role in the adaptive immune response and are crucial for controlling and eradicating viral infections [21]. CD8^+^ T cells, or cytotoxic T cells, directly eliminate cells infected with viruses by recognizing viral peptides presented on major histocompatibility complex class I molecules [22]. CD4^+^ T cells, or helper T cells, support this process by secreting cytokines that enhance overall immune responses, including the activation of B cells and CD8^+^ T cells [23]. The activation of inflammasomes notably impacts T cell responses [11], influencing the cytokine milieu which, in turn, affects T cell recruitment to the site of infection [24]. This recruitment is vital for mounting a robust inflammatory response to combat IAV infections [25].

Toll-like receptors (TLRs) also play an integral role in recognizing structural components of microbes and initiating immune responses [26]. In the context of influenza, TLR1 and TLR2 on the cell surface detect viral proteins, while TLR3, located in the endoplasmic reticulum, recognizes viral RNA [25,27]. This recognition triggers the production of cytokines that are vital for antiviral defense [28]. The crosstalk between TLRs and inflammasomes significantly enhances the immune response to IAV infections [6,24,29].

Additionally, the programmed cell death protein 1 (PD-1) and its ligand PD-L1, which are part of the immune checkpoint pathway [30], are important in regulating immune responses to prevent autoimmunity and manage immune tolerance [31]. PD-1 is expressed on T cells, B cells, and some myeloid cells, while PD-L1 is expressed on many cells, including immune cells and, importantly, in some cases, on cells infected by viruses such as influenza. During IAV infections, the interaction between PD-1 and PD-L1 can modulate the immune response, potentially reducing tissue damage by modulating T cell activity [32,33].

Epidemiological research has identified specific populations at increased risk of severe outcomes from influenza infections, including older adults, young children, individuals with obesity, pregnant women, indigenous populations, smokers, and those with underlying health conditions [3,34,35]. Notably, in the United States, the influenza- and pneumonia-attributed mortality rate within the first year following a lung cancer diagnosis (4.39%) is significantly higher than that of patients with other cancers (1.88%) [36]. However, to the best of our knowledge, the mechanism by which lung cancer impacts the severity of H3N2 IAV infection has not been well documented. Understanding how these risk factors influence the pathophysiology of influenza is crucial for developing targeted interventions. This study aims to investigate the effect of donor characteristics, particularly a history of lung cancer, on cytokine responses mediated through the inflammasome pathway during the acute phase of H3N2 IAV infection, using a human lung ex vivo culture model. We hypothesize that H3N2 IAV infection triggers distinct cellular responses and cytokine secretion profiles across different donor subgroups in ex vivo lung tissue cultures.

## 2. Results

### 2.1. Clinical Characteristics of Donors

This cross-sectional study involved 31 donors, consisting of 14 men and 17 women, with their detailed characteristics summarized in Table 1. Continuous variables were evaluated for normal distribution using the Shapiro–Wilk test. Variables that conformed to a normal distribution are presented as means ± standard deviations, whereas non-normally distributed variables are reported as medians with interquartile ranges.

The mean age of the participants was 61.2 years, and the median body mass index (BMI) was 23.9 kg/m^2^. Among the participants, the prevalence of overweight/obesity was 48% (*n* = 15), that of lung cancer was 55% (*n* = 17), that of other cancers was 29% (*n* = 9), that of hypertension was 19% (*n* = 6), that of diabetes mellitus was 13% (*n* = 4), and that of cigarette smoking (current/former) was 23% (*n* = 7). Statistical tests confirmed that the distribution of clinical characteristics between the donors with lung cancer and those without lung cancer did not differ significantly (all *p*-values ≥ 0.05).

### 2.2. Cytokine Levels in Culture Supernatants

Cytokine levels were quantified by flow cytometry. The percentage difference in cytokine levels between the H3N2 IAV infection group and the mock infection group was calculated using the following formula:

$$\mathrm{Percentage}\;\mathrm{difference}=\frac{\mathrm{Value}\;\mathrm{for}\;H3N2\;\mathrm{IAV}\;\mathrm{infection}\;\mathrm{group}-\mathrm{Value}\;\mathrm{for}\;\mathrm{mock}\;\mathrm{infection}\;\mathrm{group}}{\mathrm{Value}\;\mathrm{for}\;H3N2\;\mathrm{IAV}\;\mathrm{infection}\;\mathrm{group}+\;\mathrm{Value}\;\mathrm{for}\;\mathrm{mock}\;\mathrm{infection}\;\mathrm{group}}\times 100.$$

At 24 h post-ex vivo infection, the median percentage differences in cytokine levels were as follows (Table 2): IL-2 and IL-4 both showed no change at 0%; IL-10 exhibited a significant increase at 104%; TNF-α at 167%; and IFN-γ at 197%. The mean percentage difference for IL-6 was calculated at 45.3%. The distribution of these cytokine differences did not significantly vary between donors with lung cancer and those without (all *p*-values ≥ 0.05).

### 2.3. Molecular Expressions Related to H3N2 IAV Infection

The mean percentage differences in H3N2 nucleoprotein (NP) expression were 57.7% in epithelial cells and 55.5% in macrophages (Table 3). The mean percentage differences in TLR1 expression were 3.4% on epithelial cells and 9.0% on macrophages, while TLR2 expression showed a mean percentage difference of 14.7% on epithelial cells and −11.7% on macrophages. TLR3 expression differences were 14.7% in epithelial cells and 12.3% in macrophages. PD-L1 expression exhibited mean percentage differences of 27.4% on epithelial cells and 81.0% on macrophages. In terms of PD-1 expression, the mean percentage differences were 6.7% on CD3^+^/CD4^+^ T cells and −5.1% on CD3^+^/CD8^+^ T cells. Furthermore, the distribution of these molecular expressions did not significantly differ between donors with and without lung cancer (all *p*-values ≥ 0.05).

### 2.4. Correlations between Clinical Variables of Donors and Percentage Differences in Cytokine and Molecular Expressions

Table 4 illustrates significant correlations between clinical variables of donors and percentage differences in cytokine and molecular expressions following H3N2 IAV infection. Age positively correlated with TLR-2 expression on macrophages, but negatively with IL-4, IL-6, and TNF-α levels. BMI was positively related to NP expression in macrophages yet negatively associated with IFN-γ levels. Diabetes mellitus positively influenced TLR-2 expression on macrophages but adversely affected IL-6 and TNF-α levels. Cigarette smoking was positively associated with IL-2 levels.

### 2.5. Associations between Percentage Differences in NP Expression, Secreted Cytokine Levels, and Molecular Expression

**Figure 1:**
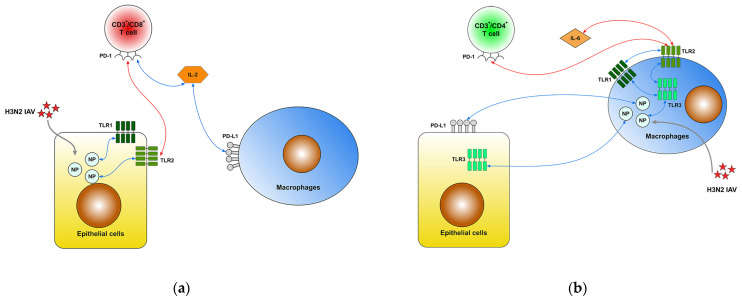
Correlations between cytokine levels and molecular expressions post-H3N2 infection. (**a**) This panel illustrates the significant correlations between percentage differences in NP expression in epithelial cells, molecular markers, and secreted cytokine levels 24 h after ex vivo infection with H3N2 influenza A virus. (**b**) This panel highlights the significant correlations between percentage differences in NP expression in macrophages, molecular markers, and secreted cytokine levels following H3N2 infection. The depicted correlations are determined using Pearson tests for normally distributed variables and the Spearman correlation for non-normally distributed variables. Positive correlations are indicated by blue lines, while negative associations are shown in red. Only correlations with two-sided *p*-values less than 0.05 are illustrated, highlighting statistically significant relationships that may impact the immune response to H3N2 infection. Abbreviations: IL: interleukin; NP: nucleoprotein; PD-1: programmed cell death protein 1; PD-L1: programmed cell death ligand 1; TLR: Toll-like receptor.

Figure 1 illustrates the relationships between percentage differences in NP expression in epithelial cells or macrophages, secreted cytokine levels, and molecular expressions following H3N2 IAV infection, 24 h post-ex vivo infection.

Positive associations were observed between the percentage difference in NP expression in epithelial cells and TL1 and TLR2 expression on the same cell type (Figure 1a). This relationship inversely correlated with the percentage difference in PD-1 expression on CD3^+^/CD8^+^ T cells, which was, in turn, positively associated with changes in secreted IL-2 levels. Additionally, IL-2 levels showed a positive correlation with PD-L1 expression on macrophages.

Moreover, the percentage difference in NP expression in macrophages was positively related to percentage differences in PD-L1 expression on epithelial cells and TLR3 expression in both epithelial cells and macrophages (Figure 1b). This upregulation in macrophages was also positively associated with TLR1 and TLR2 expressions. In contrast, the percentage difference in TLR2 expression on macrophages negatively correlated with PD-1 expression on CD3^+^/CD4^+^ T cells and secreted IL-6 levels.

Additionally, IFN-γ levels were associated with IL-10 and TNF-α levels.

### 2.6. Independent Donors’ Characteristics Affecting Percentage Differences in Secreted Cytokine Levels

Multiple linear regression models with forward selection were used to identify factors independently associated with the percentage differences in secreted cytokine levels (Table 5). The analysis revealed the following significant associations:

1. IL-2 levels: Cigarette smoking had a significant influence on IL-2 levels, with a regression coefficient of 123.00 (*p* < 0.001). Additionally, the percentage difference in PD-L1 expression on macrophages was associated with IL-2 levels (regression coefficient = 0.40; *p* = 0.02). The model explained 45% of the variance in IL-2 levels (adjusted *R*^2^ = 0.45).
2. IL-4 levels: age was independently associated with the percentage difference in secreted IL-4 levels (regression coefficient = −2.04; *p* = 0.01), explaining 18% of the variance.
3. IL-6 levels: diabetes mellitus was a significant predictor of IL-6 levels (regression coefficient = −90.57; *p* = 0.03), with the model accounting for 13% of the variance.
4. IL-10 levels: no variables of interest were found to be significantly associated with the percentage difference in secreted IL-10 levels.
5. TNF-α levels: both diabetes mellitus (regression coefficient = −183.51; *p* < 0.001) and the percentage difference in PD-1 expression on CD3^+^/CD4^+^ T cells (regression coefficient = −0.42; *p* = 0.03) were independently associated with TNF-α levels, explaining 52% of the variance.
6. IFN-γ levels: BMI was independently associated with IFN-γ levels (regression coefficient = −12.68; *p* = 0.004), accounting for 22% of the variance.

## 3. Discussion

Our study explores the intricate interactions between donor characteristics and acute cytokine responses during influenza infection. Notably, a history of lung cancer did not significantly alter cytokine responses or molecular expression in the ex vivo model. This finding suggests that the poor prognosis often associated with IAV infection in lung cancer patients may not be directly tied to the TCR signaling pathway or the PD-1/PD-L1 pathway during the acute phase. Instead, other factors, such as tumor microenvironment dynamics or immunosuppressive mechanisms, may play a more critical role in disease severity.

TLRs are critical players in both innate immunity and tumor biology. TLRs expressed on epithelial and immune cells can either promote or inhibit tumor progression, depending on the context [37]. The balance of these opposing roles is crucial in defining the lung’s immune environment [38]. We hypothesize that the diverse roles of TLRs in different cell types may also modulate the immune response to H3N2 infection, balancing pro- and anti-inflammatory signals. Lung cancer overexpresses PD-L1, allowing cancer cells to evade immune detection by suppressing T cell activation [39]. However, in our study, both lung cancer and non-lung cancer donors exhibited comparable CD3^+^/CD8^+^ T cell suppression during acute IAV infection, highlighting the need for further investigation into the precise mechanisms by which lung cancer exacerbates IAV infection severity.

Environmental factors, such as cigarette smoking, significantly impacted cytokine responses, particularly IL-2 levels. Smoking-induced increases in IL-2 were positively correlated with PD-L1 expression on macrophages, suggesting an altered immune regulation in smokers. Smoking alters both innate and adaptive immune responses, often leading to an enhanced pro-inflammatory profile [40]. This dysregulation may result in tissue damage and worse disease outcomes, even if virological responses are unaffected. IL-2, primarily produced by activated CD4^+^ T cells, plays a pivotal role in T cell proliferation, activation, and antiviral defense [41,42]. Our findings emphasize the importance of IL-2 in modulating immune responses, especially in the context of increased PD-L1 expression, which may impair T cell function via the PD-1/PD-L1 pathway [43,44].

In smokers, IL-2 production is often suppressed systemically; yet, our ex vivo model revealed increased IL-2 secretion in response to H3N2 infection. This discrepancy may reflect compensatory mechanisms or altered signaling pathways induced by smoking, potentially leading to a heightened risk of a cytokine storm and accelerated T cell exhaustion [45,46]. The interplay between smoking, IL-2 dynamics, and influenza pathogenesis warrants further exploration, particularly concerning long-term immune dysregulation and disease outcomes.

There are age-related declines in IL-4 production, primarily from CD4^+^ [47,48]. IL-4 is essential for regulating T cell differentiation and response, which is critical during IAV infection [49]. Our findings, showing a decline in IL-4 production with age, suggest that older adults may be at higher risk of poorer influenza outcomes due to reduced T cell-mediated immune responses. This is particularly important in the context of macrophage function, as IL-4 helps prevent immunosenescence and maintain macrophage efficacy in aged individuals [50].

IL-6, secreted by macrophages in response to PAMPs, is a multifunctional cytokine that bridges innate and adaptive immunity [51]. While elevated IL-6 levels are commonly seen in clinical settings, particularly in diabetic patients, our ex vivo model revealed a counterintuitive reduction in IL-6 production in diabetic samples. This may be attributed to diabetes-associated immune suppression, wherein specific immune functions, such as cytokine production, are impaired despite systemic inflammation [52,53]. The isolated ex vivo environment likely exposes this immunosuppression more clearly, showing reduced IL-6 production as a direct cellular response. Additionally, ex vivo culture conditions, including glucose levels, may not fully replicate the hyperglycemic state typical of diabetes, potentially influencing IL-6 output [54].

IL-10, an anti-inflammatory cytokine, plays a key role in limiting inflammation during infections by inhibiting pro-inflammatory cytokine production and reducing T cell activation [55,56,57]. Although we observed increased IL-10 levels following H3N2 infection, no direct correlations between IL-10 levels and molecular expression were found. However, IL-10 was positively associated with IFN-γ production, suggesting a regulatory balance between these cytokines. IL-10 can activate T cells to produce IFN-γ, enhancing immune responses while simultaneously preventing excessive tissue damage [58,59]. This intricate balance between inflammation and immune regulation is critical during influenza infection to prevent lung injury while maintaining effective viral clearance.

TNF-α, a pro-inflammatory cytokine crucial for antiviral defense, is primarily produced by macrophages and T cells [60,61]. While essential for viral clearance, TNF-α overproduction can lead to excessive inflammation, contributing to conditions like ARDS during severe influenza infections [62]. Our study showed that diabetes suppressed TNF-α responses, which may impair viral clearance and worsen disease outcomes by perpetuating immune dysfunction [63,64]. The negative association between TNF-α and PD-1 expression on CD3^+^/CD4^+^ T cells further supports the notion that diabetes-induced immune alterations could diminish the T cell response, potentially exacerbating viral infections.

IFN-γ, a key cytokine in viral defense, is crucial for both innate and adaptive immunity [65]. It enhances macrophage activity, promotes cytotoxic T cell function, and regulates cytokine production [66,67]. We found that IFN-γ modulates the immune response by influencing the production of cytokines such as TNF-α and IL-10 and regulating immune cell functions, thus ensuring a balanced response that effectively controls viral replication without causing excessive tissue damage [68,69]. Obesity is linked to reduced IFN-γ production, leading to impaired antiviral responses and increased susceptibility to severe influenza outcomes [70,71,72]. In our study, a higher BMI correlated with lower IFN-γ levels, highlighting the detrimental impact of obesity on immune function and influenza prognosis.

Our study highlights the complex regulatory network of immunological markers in response to ex vivo H3N2 IAV infection, emphasizing their role in antiviral defense. Notably, we observed an upregulation of TLR2 expression on lung epithelial cells (Figure 1a), which aligns with existing research showing that TLR2 plays a key role in recognizing viral components and initiating protective immune responses [73]. TLR2 activation triggers downstream signaling via MyD88, leading to the activation of NF-κB and subsequent production of pro-inflammatory cytokines and interferons [74], which are crucial for antiviral immunity. Interestingly, we found an inverse relationship between NP and TLR2 expression on epithelial cells and PD-1 expression on CD3^+^/CD8^+^ T cells, suggesting that enhanced TLR2-driven innate responses may simultaneously shape adaptive immunity. This could involve IL-2, which is vital for the activation and expansion of T cells, and PD-1, a checkpoint molecule that regulates T cell exhaustion [75]. The positive correlation between IL-2 levels and PD-L1 expression on macrophages further emphasizes the balance between activation and immune suppression, as PD-L1 is typically associated with inhibiting T cell responses to prevent overactive inflammation [76], highlighting how innate responses may influence adaptive immunity.

In macrophages, we observed a coordinated upregulation of TLR2, TLR3, and TLR1 expression (Figure 1b), consistent with macrophages’ role in recognizing PAMPs through multiple TLRs to mount a robust immune defense [27]. TLR3, in particular, detects viral double-stranded RNA, activating the TRIF pathway, which leads to the production of type I interferons and pro-inflammatory cytokines [77]. This upregulation of multiple TLRs suggests a strategic amplification of the macrophages’ capacity to respond to viral infection. Furthermore, higher TLR2 expression in macrophages was associated with reduced PD-1 expression on CD3^+^/CD4^+^ T cells and lower IL-6 levels, suggesting a finely tuned balance where macrophage-driven innate immunity may limit T cell suppression and excessive inflammation. This could be a protective mechanism to prevent cytokine storms, often seen in severe IAV infections. In contrast, increased NP expression in macrophages was linked to elevated PD-L1 and TLR3 expressions in epithelial cells, which may engage PD-1 on T cells in suppressing hyperactive immune responses, preventing immune-mediated tissue damage. Elevated TLR3 expression in epithelial cells, however, promoted cytokine secretion, including IL-6 and IL-8, reflecting its role in driving inflammatory responses [78]. These findings indicate that TLR3 activation can both aid in antiviral defense and contribute to inflammation, highlighting the dual role of TLRs in infection.

Inflammasome-induced cytokines such as IL-1β, IL-6, and transforming growth factor-β (TGF-β) can drive the differentiation of CD4^+^ T cell precursors into Th17 cells, known for producing IL-17 in response to pathogens and contributing to autoimmune and chronic inflammatory diseases [79,80]. In the presence of allergens and IL-4, Th17 cells may exacerbate inflammation in allergic conditions by promoting IgE production [81,82]. Conversely, TGF-β signaling can induce a protective, anti-inflammatory phenotype in Th17 cells through IL-10 production [83]. Additionally, IAV infection stimulates pulmonary invariant natural killer cells to produce IFN-γ and IL-22, enhancing mucosal immunity. While IL-22, a Th17-related cytokine, aids in protecting airway epithelial cells from mortality, it does not affect viral replication [84]. However, persistent viral infections can provoke sustained Th17 responses, leading to chronic inflammation and potentially fostering tumor growth and progression [85]. These findings highlight the complex role of Th17 cells in acute H3N2 infection and underscore the need for further research into their impact.

This study has several limitations that should be considered when interpreting the findings. Firstly, the use of a small sample size and specific selection criteria may limit the generalizability of the results to a broader population. Secondly, as this was an ex vivo study, the results may not fully translate to in vivo or clinical settings, potentially affecting their practical applicability. Additionally, this study did not fully account for potential confounding variables that could influence the outcomes. Important factors such as vitamin D levels and microbiota composition, which are known to regulate the host immune system and play roles in allergic and autoimmune diseases, were not investigated [86,87]. Moreover, physiological processes such as aging and sex differences could impact vitamin D levels and the diversity of the microbiome, further complicating the interpretation [88]. The findings may also not be directly applicable across different demographic or geographical settings due to varying external conditions. While human studies can provide insights into real-world implications of these interactions, ex vivo studies offer a more detailed understanding of the underlying cellular mechanisms. Therefore, integrating insights from both types of research is essential for developing effective therapeutic and preventive strategies tailored to diverse populations and individual characteristics.

## 4. Materials and Methods

### 4.1. Study Design

The study protocol for this cross-sectional investigation was approved by the Institutional Review Board at Chang Gung Medical Foundation, Taoyuan, Taiwan (Approval No: 201702269B0C501), in compliance with the ethical standards of the 1975 Declaration of Helsinki. All participants provided written informed consent prior to inclusion. Details of the original study protocol have been previously described [44].

This study included 31 patients who underwent video-assisted thoracoscopic surgery for suspected lung cancer at Chang Gung Memorial Hospital, Linkou Main Branch, Taoyuan, Taiwan, between 1 August 2018 and 31 December 2019. Eligibility criteria were age over 20 years, undergoing surgery for suspected lung cancer, and willingness to provide written informed consent. Exclusion criteria included a recent respiratory tract viral infection within the past two weeks, a known history of chronic viral infections such as HIV, or a diagnosis of interstitial lung disease, severe chronic obstructive pulmonary disease, pulmonary fibrosis, or severe lung infectious diseases.

Baseline clinical characteristics recorded for all participants included age, sex, BMI, history of cancer (lung or other types), medical comorbidities (e.g., hypertension, diabetes, hyperlipidemia, coronary artery disease), and smoking history.

### 4.2. Lung Explant Tissue Processing and Ex Vivo Infection with H3N2 IAV

Human parenchymal tissue samples, each measuring at least 1 cm^3^ and obtained from areas distal to resection margins and any visible pathology, were carefully excised from the lobe. These samples were initially stored in Roswell Park Memorial Institute (RPMI) 1640 medium, supplemented with 1% penicillin–streptomycin (Life Technologies, Paisley, Renfrewshire, UK) and 1% gentamicin (GE Healthcare, Little Chalfont, Chalfont St Giles, UK). After storage, the tissues were sectioned into 1 mm^3^ pieces, with three sections placed into each well of a 24-well flat-bottomed culture plate. The tissue sections were washed three times with Dulbecco’s phosphate-buffered saline (PBS) (Sigma-Aldrich, Poole, Dorset, UK) and once with RPMI 1640 medium. Following the washes, the tissues were incubated overnight at 37 °C in a 5% CO_2_ atmosphere in RPMI 1640 medium supplemented with 1% penicillin–streptomycin and 1% gentamicin.

Subsequently, resected lung tissues were infected with the seasonal H3N2 IAV strain A/Texas/50/2012 or mock-infected, following established protocols [44,89,90]. After removing the old culture medium, the tissues were immersed in serum-free RPMI medium supplemented with 100 U/mL of penicillin, 100 μg/mL of streptomycin, 2 mM of L-glutamine, and 250 ng/mL of fungizone. The lung explants were then exposed to either 1 × 10⁶ plaque-forming units per milliliter of H3N2 IAV or no virus, followed by a 2 h incubation at 37 °C in a 5% CO_2_ atmosphere. After incubation, the tissues were washed three times with RPMI medium and incubated for an additional 22 h in the same serum-free medium.

Following this period, the culture medium was centrifuged at 2000 rpm at 4 °C for 5 min, and the supernatants were carefully collected and immediately frozen at −80 °C for future cytokine analysis.

The explanted lung tissues were further processed by digestion with 150 U/mL of type I collagenase and 10 U/mL of type IIA elastase (Sigma Chemical Co., St. Louis, MO, USA) at 37 °C for 15 min, as previously described [91]. After digestion, the tissues were mechanically disrupted using a tissue grinder and passed through a 35 μm cell sieve to optimize cell yield. The dissociated tissue was then washed once with PBS at 400 × g for 5 min. The isolated cells were analyzed via flow cytometry to assess immune cell populations and relevant markers.

### 4.3. Measurement of Secreted Cytokine Levels by Flow Cytometry

Cytokine concentrations for IL-2, IL-4, IL-6, IL-10, TNF-α, and IFN-γ in the culture supernatants were determined using a cytometric bead array technique. This assay was performed with the Human Soluble Protein Flex Set System (BD Biosciences, San Jose, CA, USA). Phycoerythrin-conjugated antibodies specific to the target cytokines were added to the supernatants, allowing the antibodies to bind their respective cytokines. These complexes were then stained according to the manufacturer’s protocol. The cytokine concentrations were quantified via flow cytometry.

### 4.4. Measurement of IAV Infection-Related Molecular Expressions by Flow Cytometry

Flow cytometry was used to evaluate molecular biomarkers associated with IAV infection, following methodologies from previous research. Cells isolated from lung tissues were resuspended in BD Pharmingen stain buffer (Becton Dickinson Biosciences, catalog No: 554656), supplemented with heat-inactivated fetal bovine serum (FBS) proteins. Depending on the protocol, the cells were incubated either on ice in the dark or at 56 °C in the dark for 30 min. The staining of specific molecular markers was performed using commercially available kits, following the manufacturer’s instructions.

To reduce nonspecific antibody binding—especially in lung macrophages—a post-fixation and permeabilization step was included, followed by a 30 min incubation in FBS-containing stain buffer on ice, shielded from light, to block Fc receptors as outlined in [17].

During the staining process, a fluorescently labeled antibody specific to the molecule of interest was added, with an isotype-matched antibody from the same manufacturer serving as a specificity control. Molecular expressions were analyzed using the FACSAria^TM^ IIu Cell Sorter and FACSDiva^TM^ software v5.0.3 (Becton Dickinson Biosciences). An unstained singlet population from the digested tissue was used to establish a baseline, and dead cells were excluded using Fixable Viability Stain 700 (Becton Dickinson Biosciences, catalog No: 564997), enabling accurate analysis of IAV-related molecular expressions in lung tissue samples.

#### 4.4.1. Identification of Epithelial Cells, Macrophages, CD3^+^/CD4^+^ T Cells, and CD3^+^/CD8^+^ T Cells

**Figure 2:**
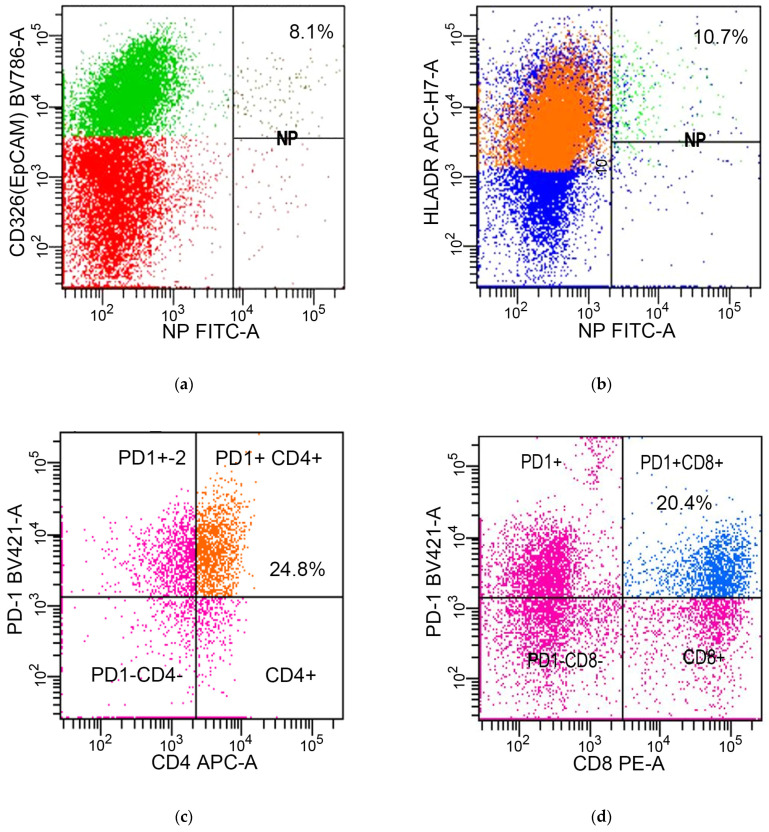
Flow cytometry analysis of molecular expressions in various cell populations during H3N2 infection. (**a**) Expressions of influenza A virus NP (green) in EpCAM^+^ epithelial cells; (**b**) expressions of influenza A virus NP (green) in HLA-DR^+^ macrophages; (**c**) expressions of PD-1 (orange) on CD3^+^/CD4^+^ T cells; (**d**) expressions of PD-1 (blue) on CD3^+^/CD8^+^ T cells. Abbreviations: EpCAM, epithelial cell adhesion molecule; HLA, human leukocyte antigen; NP, nucleoprotein; PD-1, programmed death 1.

In this study, the detection and analysis of specific cell types were central to understanding the immune landscape in response to H3N2 IAV infection. The cell types identified included CD45^−^/EpCAM^+^ epithelial cells (Figure 2a), CD45^+^/HLA-DR^+^ macrophages (Figure 2b), CD45^+^/CD3^+^/CD4^+^ T cells (Figure 2c), and CD45^+^/CD3^+^/CD8^+^ T cells (Figure 2d), as cited in previous research. Specific antibodies were used to target the markers CD45, EpCAM, HLA-DR, CD3, CD4, and CD8. These included peridinin chlorophyll protein-Cyanine5.5 conjugated mouse anti-human CD45 antibody (catalog no. 564106), brilliant violet 786 conjugated mouse anti-human CD326 (EpCAM) antibody (catalog no. 565685), allophycocyanin-H7 conjugated mouse anti-human HLA-DR antibody (catalog no. 568570), fluorescein isothiocyanate (FITC)-conjugated mouse anti-human CD3 antibody (catalog no. 555332), phycoerythrin conjugated mouse anti-human CD4 antibody (catalog no. 561843), and phycoerythrin conjugated mouse anti-human CD8 antibody (catalog no. 560959), all sourced from Becton Dickinson Biosciences, San Jose, CA, USA. These fluorescently labeled antibodies were utilized as per the manufacturer’s guidelines to accurately assess the surface expression of these markers on lung tissue cells.

#### 4.4.2. Intracellular Viral NP Expression

This section of this study concentrates on the intracellular expression of the H3N2 IAV NP in epithelial cells and macrophages, moving away from T cells, in line with previously established research [92,93,94,95]. To facilitate the intracellular staining of viral NP, the BD Cytofix/Cytoperm fixation/permeabilization solution (catalog no. 554714) was utilized. After preparation of the cells, an FITC-conjugated anti-IAV NP monoclonal antibody (catalog no. MA1-7322, Thermo Fisher Scientific, Waltham, MA, USA) was applied to detect the NP (Figure 2a,b), following the specified protocols for accurate and effective labeling of the targeted viral protein [96].

#### 4.4.3. Measurement of TLRs

**Figure 3:**
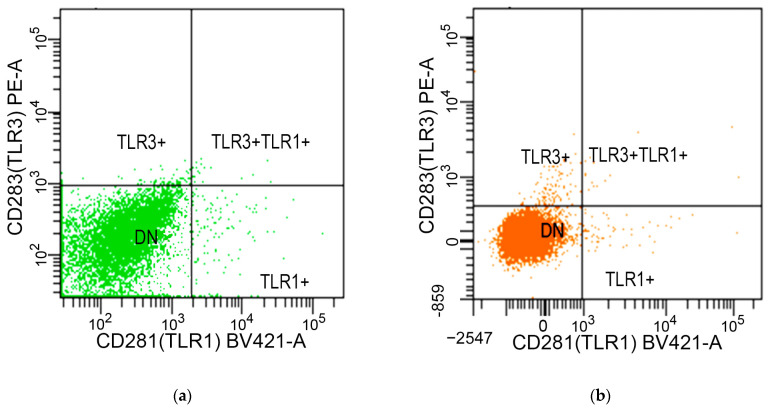

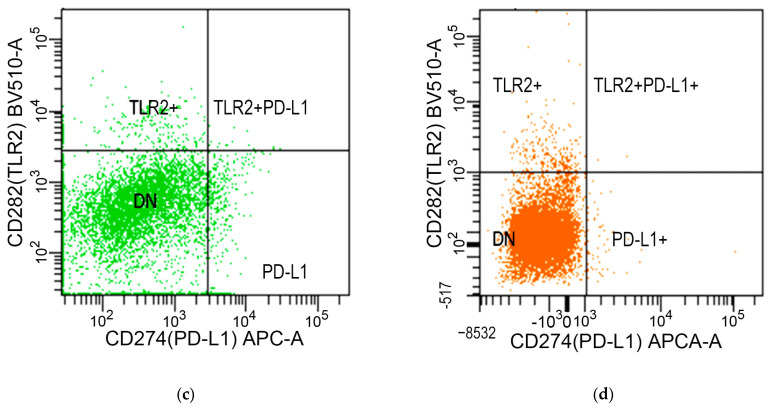
Flow cytometry analysis of TLR and PD-L1 expressions in cell populations during H3N2 infection. (**a**) Expressions of TLR1 (green) and/or TLR3 (green) on epithelial cells; (**b**) expressions of TLR1 (orange) and/or TLR3 (orange) on macrophages; (**c**) expressions of TLR2 (green) and/or PD-L1 (green) on epithelial cells; (**d**) expressions of TLR2 (orange) and/or PD-L1 (orange) on macrophages. Abbreviations: DN, double negative; PD-L1, programmed death-ligand 1; TLR, Toll-like receptor.

To evaluate the expression of TLRs relevant to immune response mechanisms, specific antibodies were utilized for TLR detection. Brilliant violet 421-conjugated mouse monoclonal antibodies were used for TLR1 (CD281) (catalog no. 566430) (Figure 3a,b) and TLR2 (CD282) (catalog no. 565350) (Figure 3c,d). Additionally, a phycoerythrin-conjugated mouse monoclonal antibody was employed for TLR3 (CD283) (catalog no. 565984) (Figure 3a,b). These antibodies facilitated the specific staining and subsequent quantification of TLRs on selected cells, adhering closely to the protocols provided by the manufacturer.

#### 4.4.4. Measurement of PD-1/PD-L1 Pathways

This study assessed the expression of the PD-1/PD-L1 pathway components in various immune and epithelial cell types. PD-1 expressions were measured on CD3^+^/CD4^+^ T cells and CD3^+^/CD8^+^ T cells using brilliant violet 421-conjugated mouse anti-human PD-1 (CD279) antibody (catalog no. 565942) (Figure 2c,d). For the assessment of PD-L1 expressions on epithelial cells and macrophages, phycoerythrin-conjugated mouse anti-human PD-L1 (CD274) antibody (catalog no. 568722) was utilized (Figure 3c,d). Both staining processes were conducted strictly according to the manufacturer’s guidelines, ensuring precise and reliable detection of these key immunological markers in the study samples.

### 4.5. Sample Size Estimation

The sample size for this study was calculated based on the methodologies described in our previous research [44]. We estimated that a total of 30 donors would be needed to effectively differentiate viral expression levels at 24 h post infection across different age groups. This estimation was derived using a two-tailed Mann–Whitney *U* test, with a power of 80% and a type I error rate of 0.05.

### 4.6. Statistical Analysis

Continuous variables were assessed for normality using the Shapiro–Wilk test. Descriptive statistics for these variables include means, standard deviations, medians, and interquartile ranges, while categorical variables are reported as counts and proportions. For between-group comparisons of continuous variables, independent-samples *t* tests were used for normally distributed data, and Mann–Whitney *U* tests were applied for non-normally distributed data. Fisher’s exact test was used to compare two independent categorical variables.

Correlation analyses were conducted using Pearson’s correlation test for normally distributed continuous variables, Spearman’s correlation test for non-normally distributed variables, or the Point-Biserial correlation for relationships between continuous and binary variables. Multivariable linear regression models with forward selection were employed to identify independent variables affecting percentage differences in cytokine levels, with a significance threshold of *F* < 0.05. The variance inflation factor was calculated for each predictor to assess multicollinearity, and any variable with a variance inflation factor of 5 or higher was excluded to recalibrate the regression models.

Statistical analyses were performed using G*Power 3.1.9.2 (Heinrich-Heine University, Düsseldorf, Germany) and SPSS (version 29; International Business Machines Corp., Armonk, NY, USA). A two-sided *p*-value of less than 0.05 was considered statistically significant.

## 5. Conclusions

In summary, this study reveals the complex interactions between donor characteristics, immune responses, and molecular pathways during H3N2 IAV infection using an ex vivo lung tissue model. A history of lung cancer did not significantly affect cytokine responses, suggesting that other factors, such as tumor microenvironment dynamics, may contribute to infection severity. Key immune pathways involving TLR2, TLR3, and PD-1/PD-L1 were shown to modulate the balance between innate and adaptive immunity, with smoking, aging, diabetes, and obesity also impacting critical cytokine responses. These findings offer valuable insights into immune regulation in different patient subgroups and underscore the importance of developing targeted therapeutic strategies for high-risk populations.

## Figures and Tables

**Table 1:** Clinical characteristics of donors.

Variables	Overall	Lung Cancer (+)	Lung Cancer (−)	*p*-Value ^1^
Number	31 (100)	17 (55)	14 (45)	
Age, year	61.2 ± 11.0	63.5 ± 10.0	58.4 ± 11.9	0.21
BMI, kg/m^2^	23.9 (22.2 to 25.3)	24.5 (22.7 to 27.2)	22.7 (21.9 to 24.6)	0.20
Overweight/obesity, *n* (%)	15 (48)	10 (59)	5 (36)	0.29
Other cancer ^2^, *n* (%)	9 (29)	4 (24)	5 (36)	0.69
Hypertension, *n* (%)	6 (19)	4 (24)	2 (14)	0.66
Diabetes mellitus, *n* (%)	4 (13)	2 (12)	2 (14)	>0.99
Cigarette smoking (current/former), *n* (%)	7 (23)	2 (12)	5 (36)	0.20

Data are presented as means ± standard deviations for normally distributed variables, medians (interquartile ranges) for non-normally distributed variables, and number (%) for categorical variables. Abbreviations: BMI: body mass index; (+): positive; (−): negative. ^1^ Independent-samples *t* test was used for normally distributed variables, independent-samples Mann–Whitney *U* test for non-normally distributed variables, and Fisher’s exact test for categorical variables. ^2^ Other cancers include laryngeal (*n* = 1), gastric (*n* = 1), prostate (*n* = 2), cervical (*n* = 2), rectal (*n* = 2), liver (*n* = 1), and thyroid (*n* = 1) cancers.

**Table 2:** Cytokine responses to H3N2 influenza A virus infections.

Variables	Overall	Lung Cancer (+)	Lung Cancer (−)	*p*-Value ^1^
Percentage Difference in IL-2 secretion	0 (0 to 186)	7 (0 to 195)	0 (0 to 184)	0.63
Percentage Difference in IL-4 secretion	0 (0 to 0)	0 (0 to 0)	0 (0 to 0)	0.38
Percentage Difference in IL-6 secretion	45.3 ± 76.8	45.3 ± 91.5	45.4 ± 57.4	>0.99
Percentage Difference in IL-10 secretion	104 (0 to 191)	114 (−27 to 192)	93 (0 to 177)	0.95
Percentage Difference in TNF-α secretion	167 (122 to 194)	167 (116 to 190)	167 (115 to 199)	0.65
Percentage Difference in IFN-γ secretion	197 (188 to 199)	197 (78 to 199)	198 (195 to 200)	0.11

Data are presented as means ± standard deviations for normally distributed variables and medians (interquartile ranges) for non-normally distributed variables. Abbreviations: IFN-γ: interferon-gamma; IL: interleukin; TNF-α: tumor necrosis factor-alpha; (+): positive; (−): negative. ^1^ Independent-samples *t* test was used for normally distributed variables and independent-samples Mann–Whitney *U* test for non-normally distributed variables.

**Table 3:** Molecular expression responses to H3N2 influenza A virus infections.

Variables	Overall	Lung Cancer (+)	Lung Cancer (−)	*p*-Value ^1^
Percentage Difference in Intracellular H3N2 NP Expression				
Epithelial Cells	57.7 ± 96.0	56.8 ± 92.7	58.8 ± 103.5	0.96
Macrophages	55.5 ± 80.1	55.4 ± 69.3	55.7 ± 94.3	0.99
Percentage Difference in Surface TLR1 Expression				
Epithelial Cells	3.4 ± 80.4	−16.7 ± 84.9	27.9 ± 69.8	0.13
Macrophages	9.0 ± 83.8	−17.4 ± 78.1	41.1 ± 41.7	0.05
Percentage Difference in Surface TLR2 Expression				
Epithelial Cells	14.7 ± 76.4	3.0 ± 81.2	29.0 ± 70.4	0.36
Macrophages	−11.7 ± 73.5	−23.6 ± 75.7	2.7 ± 70.9	0.33
Percentage Difference in Intracellular TLR3 Expression				
Epithelial Cells	14.7 ± 51.9	12.5 ± 50.4	17.3 ± 55.4	0.80
Macrophages	12.3 ± 91.4	−3.2 ± 84.8	31.1 ± 98.6	0.31
Percentage Difference in Surface PD-L1 Expression				
Epithelial Cells	27.4 ± 80.8	18.3 ± 74.0	38.4 ± 89.9	0.50
Macrophages	81.0 ± 72.7	86.1 ± 76.2	74.9 ± 70.5	0.68
Percentage Difference in Surface PD-1 Expression				
CD3^+^/CD4^+^ T Cells	6.7 ± 58.9	7.1 ± 30.6	6.2 ± 82.8	0.97
CD3^+^/CD8^+^ T Cells	−5.1 ± 35.4	−2.8 ± 38.8	−7.8 ± 32.0	0.70

Data are presented as means ± standard deviations. Abbreviations: NP: nucleoprotein; PD-1: programmed cell death protein 1; PD-L1: programmed cell death ligand 1; TLR: Toll-like receptor; (+): positive; (−): negative. ^1^ Independent-samples *t* test was used for normally distributed variables.

**Table 4:** Correlations between donors’ clinical variables and percentage differences in secreted cytokine levels and molecular expression ^1^.

Variables	Age	Man	BMI	Lung Cancer	Non-Lung Cancer	Hypertension	Diabetes Mellitus	Cigarette Smoking
Percentage Difference in Secreted Cytokine Levels in Supernatant								
IL-2	−0.11 (0.57)	0.48 (0.01)	0.10 (0.61)	−0.10 (0.58)	−0.04 (0.85)	−0.16 (0.39)	−0.22 (0.23)	**0.57 (<0.001)**
IL-4	**−0.40 (0.02)**	0.12 (0.52)	−0.19 (0.31)	−0.33 (0.08)	−0.07 (0.71)	−0.31 (0.09)	−0.04 (0.82)	−0.04 (0.82)
IL-6	**−0.37 (0.04)**	0.09 (0.64)	0.09 (0.65)	−0.01 (> 0.99)	0.23 (0.21)	0.06 (0.77)	**−0.40 (0.03)**	0.08 (0.67)
IL-10	−0.15 (0.42)	0.19 (0.30)	−0.16 (0.40)	−0.01 (0.95)	−0.16 (0.39)	−0.20 (0.28)	−0.28 (0.14)	0.07 (0.70)
TNF-α	**−0.38 (0.04)**	−0.24 (0.20)	−0.07 (0.69)	−0.09 (0.64)	0.01 (> 0.99)	0.14 (0.46)	**−0.46 (0.01)**	−0.12 (0.52)
IFN-γ	0.03 (0.87)	0.10 (0.59)	**−0.39 (0.03)**	−0.30 (0.10)	−0.11 (0.55)	−0001 (0.90)	−0.24 (0.20)	0.04 (0.85)
Percentage Difference in Intracellular H3N2 NP Expression								
Epithelial Cells	−0.13 (0.49)	−0.20 (0.28)	−0.20 (0.28)	−0.01 (0.96)	0.15 (0.44)	0.13 (0.5)	−0.33 (0.07)	−0.18 (0.33)
Macrophages	0.08 (0.67)	−0.23 (0.21)	**0.37 (0.04)**	−0.01 (> 0.99)	−0.01 (0.98)	0.22 (0.23)	0.27 (0.15)	−0.01 (0.99)
Percentage Difference in Surface TLR1 Expression								
Epithelial Cells	−0.21 (0.27)	−0.06 (0.74)	0.12 (0.51)	−0.28 (0.13)	−0.10 (0.61)	−0.02 (0.91)	0.01 (0.96)	0.15 (0.43)
Macrophages	0.13 (0.48)	−0.04 (0.85)	0.01 (0.96)	−0.35 (0.05)	0.17 (0.36)	0.12 (0.54)	0.32 (0.09)	−0.03 (0.87)
Percentage Difference in Surface TLR2 Expression								
Epithelial Cells	−0.22 (0.23)	−0.34 (0.06)	0.17 (0.35)	−0.17 (0.36)	0.01 (0.95)	0.07 (0.69)	0.07 (0.72)	−0.06 (0.75)
Macrophages	**0.37 (0.04)**	−0.12 (0.53)	0.35 (0.05)	−0.18 (0.33)	0.22 (0.25)	0.31 (0.09)	**0.39 (0.03)**	−0.03 (0.88)
Percentage Difference in Intracellular TLR3 Expression								
Epithelial Cells	−0.22 (0.23)	0.03 (0.86)	−0.16 (0.40)	−0.05 (0.80)	−0.12 (0.54)	−0.27 (0.15)	0.05 (0.81)	−0.07 (0.70)
Macrophages	0.21 (0.25)	−0.17 (0.37)	0.29 (0.12)	−0.19 (0.31)	0.07 (0.71)	0.19 (0.31)	0.32 (0.08)	0.02 (0.92)
Percentage Difference in Surface PD-L1 Expression								
Epithelial Cells	−0.07 (0.72)	0.17 (0.35)	−0.02 (0.91)	−0.13 (0.50)	0.30 (0.10)	0.06 (0.76)	0.09 (0.65)	−0.07 (0.73)
Macrophages	0.06 (0.77)	0.28 (0.13)	0.24 (0.20)	0.08 (0.68)	0.17 (0.36)	0.10 (0.61)	−0.11 (0.55)	0.11 (0.55)
Percentage Difference in Surface PD-1 Expression								
CD3^+^/CD4^+^ T Cells	−0.10 (0.61)	−0.03 (0.89)	−0.10 (0.58)	0.01 (0.97)	0.03 (0.86)	−0.07 (0.70)	−0.24 (0.19)	−0.02 (0.91)
CD3^+^/CD8^+^ T Cells	−0.09 (0.64)	0.27 (0.14)	0.14 (0.44)	0.07 (0.70)	0.01 (> 0.99)	−0.12 (0.53)	−0.05 (0.80)	−0.03 (0.89)

Data are presented as correlation coefficients (*p*-value). Abbreviations: BMI: body mass index; IFN-γ: interferon-gamma; IL: interleukin; NP: nucleoprotein; PD-1: programmed cell death protein 1; PD-L1: programmed cell death ligand 1; TLR: Toll-like receptor; TNF-α: tumor necrosis factor-alpha. ^1^ Pearson correlation tests were used for normally distributed variables, Spearman correlation for non-normally distributed variables and non-categorical variables, and Point-Biserial correlation for categorical variables with other variables. Bold values indicate statistically significant differences (two-tailed *p* < 0.05).

**Table 5:** Independent donors’ characteristics affecting percentage differences in secreted cytokine levels in response to H3N2 influenza A virus infections.

Variables	Regression Coefficient (95% CI) ^1^	*p*-Value	VIF	Adjusted *R*^2^
Percentage Difference in secreted IL-2 level				
Cigarette Smoking	123.00 (63.69 to 182.31)	<0.001	1.01	0.45
Percentage Difference in PD-L1 Expression on Macrophages	0.40 (0.06 to 0.75)	0.02	1.01	
Percentage Difference in secreted IL-4 level				
Age	−2.04 (−3.57 to −0.52)	0.01	1.00	0.18
Percentage Difference in secreted IL-6 level				
Diabetes Mellitus	−90.57 (−168.92 to −12.22)	0.03	1.00	0.13
Percentage Difference in secreted IL-10 level				
None				
Percentage Difference in secreted TNF-α level				
Diabetes Mellitus	−183.51 (−248.71 to −118.31)	<0.001	1.06	0.52
Percentage Difference in PD-1 Expression on CD3^+^/CD4^+^ T Cells	−0.42 (−0.79 to −0.04)	0.03	1.06	
Percentage Difference in secreted IFN-γ level				
BMI	−12.68 (−21.04 to −4.32)	0.004	1.00	0.22

Abbreviations: CI: confidence interval; PD-1: programmed cell death protein 1; PD-L1: programmed cell death ligand 1; VIF: variance inflation factor. ^1^ Multiple linear regression models with forward selection were employed to identify factors independently associated with the percentage differences in secreted cytokine levels.

## Data Availability

The data presented in this study are available on request from the corresponding author due to ethical reasons.

## Abbreviations

ARDS	acute respiratory distress syndrome
BMI	body mass index
DAMPs	damage-associated molecular patterns
DN	double negative
FBS	fetal bovine serum
IAV	influenza A virus
IFN-γ	interferon-gamma
IL	interleukin
NK	nature killer
NP	nucleoprotein
PAMPs	pathogen-associated molecular patterns
PD-1	programmed cell death protein 1
PD-L1	programmed death-ligand 1
TGF-β	transforming growth factor-β
TLR	Toll-like receptor
TNF-α	tumor necrosis factor-alpha
VIF	variance inflation factor
